# Development and Evaluation of a Parenting Resilience Elements Questionnaire (PREQ) Measuring Resiliency in Rearing Children with Developmental Disorders

**DOI:** 10.1371/journal.pone.0143946

**Published:** 2015-12-03

**Authors:** Kota Suzuki, Tomoka Kobayashi, Karin Moriyama, Makiko Kaga, Michio Hiratani, Kyota Watanabe, Yushiro Yamashita, Masumi Inagaki

**Affiliations:** 1 Department of Developmental Disorders, National Institute of Mental Health, National Center of Neurology and Psychiatry (NCNP), Kodaira, Tokyo, Japan; 2 Department of Pediatrics, NTT Medical Center Tokyo, Shinagawa, Tokyo, Japan; 3 Tokyo Metropolitan Medical Center EAST for children/adults with developmental disabilities, Koto, Tokyo, Japan; 4 Hiratani Child Development Clinic, Fukui, Fukui, Japan; 5 Department of Child and Adolescent Psychiatry, National Center for Global Health and Medicine, Kohnodai Hospital, Ichikawa, Chiba, Japan; 6 Department of Pediatrics and Child Health, Kurume University School of Medicine, Kurume, Fukuoka, Japan; Hamamatsu University School of Medicine, JAPAN

## Abstract

We developed a parenting resilience elements questionnaire (PREQ) measuring the degree to which mothers possess elements that aid in adapting to challenges and difficulties related to children with developmental disorders (DD). A total of 424 parents of children with DD were recruited from five medical institutes. Psychometric properties of PREQ were evaluated using data of 363 mothers of children with DD. Furthermore, multiple regression analysis was performed, predicting depressive symptoms and parenting behavior with PREQ subscales, a general health questionnaire, and the total difficulties score of a strength and difficulties questionnaire. Factor analysis revealed three reliable factors: “knowledge of the child’s characteristics,” “perceived social supports,” and “positive perceptions of parenting.” Moreover, multiple regression analysis showed that “knowledge of the child’s characteristics” was associated with parenting behavior, whereas “perceived social supports” predicted depressive symptoms; “positive perceptions of parenting” influenced both parenting behavior and depressive symptoms. These findings indicated that the PREQ may be used as a scale measuring resiliency in mothers of children with DD and is useful for evaluating their parenting ability in clinical interventions.

## Introduction

Numerous challenges and difficulties present themselves when rearing children with developmental disorders (DD), e.g., attention deficit hyperactivity disorder (ADHD), autism spectrum disorder (ASD), intellectual disability (ID), and learning disorder (LD). Furthermore, caregivers of children with DD tend to experience psychological distress that is caused by the children themselves [1, 2]. Compared with caregivers of typical developing children, caregivers of children with DD face higher risk of depression [3]. The presence of a DD is related to behavioral problems, which has been suggested as being associated with overreactive and aggressive parenting behavior by caregivers [4, 5]. These findings showed that caregivers of children with DD face adversities due to the circumstances of their children.

However, not all caregivers of children with DD present with severe psychological distress and overreactive parenting behaviors. Bebko et al. [6] reported that caregivers of children with autism experienced lesser stress from child symptoms than the caregiver’s stress level rated by professionals. Moreover, Skinner et al. [7] reported that mothers of children with IDs viewed their children as bringing about positive transformations in their life. Hastings and Taunt [8] suggested that caregivers of children with DD have positive perceptions and experiences of rearing their child. These findings suggest that most caregivers are well-adapted to challenges and difficulties associated with rearing children with DD.

Previous studies suggest that several elements contribute to positive adaptation in caregivers of children with DD. Social support has been reported to be one of these elements. Lack of social support causes higher levels of stress and depression [9]. Smith et al. [10] reported that having a larger social network was associated with an increase in well-being of mothers of individuals with ASD, whereas a negative valance of social support was associated with a decrease in well-being. The cognitive style of caregivers is also considered to affect how challenges and difficulties relating to raising children with DD impact mental health. Hastings et al. [11] reported that positive coping strategies are related to a decrease in depressive symptoms in mothers of autistic children, whereas active avoidance coping strategies was positively correlated with the psychological distress levels in such mothers. Previous studies have shown that psychological distress was influenced by the attribution of controllability of child behavior in caregivers of children with ADHD [12] and ASD [13].

It is inconvenient to evaluate the primary caregiver’s capacity for positive adaptation using multiple scales though the assessment of this capacity is useful for interventions for children with DD. We considered that factors promoting a caregiver’s adaptation can be integrated into the concept of “resilience.” Resilience refers to a process or phenomenon of positive adaptation to adversity [14]. Some questionnaires measuring resiliency have been developed for the general population [15]. The nature of the adversity or situation differed among people, such that the definition of resilience for a given group is more useful in practice rather than as a global definition [16]. Moreover, the adversity and situation of caregivers of children with DD is very different from those of the conventional resilience studies. The nature of adversity is associated with children with DD [1–5], which imply that it is continuing across their life and is changed according to development of their children. In addition, resilience should not be defined considering only mental health of caregivers. That is, elements which appear to improve mental health of caregivers but exacerbate the behavioral and mental problem of children (e.g., neglect of child-rearing) should be excluded in the construct of parenting resilience. Thus, the development of a new questionnaire was necessary for evaluating resiliency in primary caregivers of children with DD.

Qualitative studies indicate the usefulness of the construct of resilience for caregivers or families of children with DD [17–19]. Though similar elements related to resilience were reported in these studies, the model of parenting resilience [17, 20] was suitable for the aim of assessing resiliency of primary caregivers of children with DD in the Japanese population. Thus, in the present study, a parenting resilience elements questionnaire (PREQ) was developed to measure the degree to which primary caregivers possess elements relating to parenting resilience [17].

Parenting resilience was defined as the process of positive adaptation to the difficulties associated with rearing children with DD [20]. Based on this definition, we investigated the construct of parenting resilience in mothers of individuals with ASD [17]. In the study, 23 mothers of adults with ASD, who had adapted well to the challenges of rearing their child, were interviewed about their experience of child rearing from infancy to adulthood. The narrative data was analyzed using a modified grounded theory approach (M-GTA) [21]. Then, it was assumed that parenting resilience comprises twelve concepts and five categories, i.e., “a sense of motherhood,” “self-efficacy,” “knowledge of the child’s characteristics,” “perceived social supports,” and “foresight.” As the model assumed that these concepts and categories enable caregivers to cope with challenges and difficulties relating to their children, we predicted that higher PREQ scores would be associated with decrease in psychological distress levels and overreactive parenting behavior.

## Method

### Ethics Statement

The study protocol was approved by the Ethics Committee of the National Center of Neurology and Psychiatry (Protocol A2012-006, Japan). Research was conducted in accordance with the Declaration of Helsinki. Written informed consent was obtained from each parent after provision of a complete description of this study.

### Participants

A total of 424 parents of children with DD were recruited from six medical institutes in Kanto, Hokuriku, Chugoku, and Kyushu districts in Japan (response rate = 71%). Their children were diagnosed with DD—ASD, ADHD, ID, or LD—by certified child neurologists or psychiatrists at these institutes.

In the current study, only the data of mothers whose children ranged in age from 3 to 18 years were considered for analysis (n = 405). We used data of 363 mothers for factor analysis and of 313 mothers for further analysis, as missing data were excluded. Tables 1 and 2 display the demographic data of the participants and their children. Participants were asked to respond to questions about the child, or children in cases where they had several children, with DD.

**Table 1:** Demographic characteristics of participants and their children.

Variables (range and/or unit)	Mean (SD)/percentage
Age (28–54 years old)	41.58 (5.40)^a^
College graduation (%)	18.73^a^
Full or part time employment (%)	57.85^a^
Number of children (1–4)	2.02 (0.78)^a^
Birth order (1–4)	1.44 (0.67)^a^
Child's age (3–18 years old)	10.18 (3.50)^a^
Age of child’s diagnosis (1–16 years old)	6.61 (3.17)^a^
Medicated children (%)	55.92^a^
Child’s Fatherless family structure (%)	16.25^a^
Clinical range of CES-D (%)	32.69^b^
High score of PS (%)	22.36^b^

^a^ N = 363,

^b^ N = 313

CES-D: center for epidemiologic studies depression scale (cutoff score = 16)

PS: Parenting Scale (we defined the high score as +1SD above from the non-clinical sample [22]).

**Table 2:** Type of diagnosis (%).

		+LD	+ID
ADHD	25.90	4.41	1.10
ASD	42.42	1.93	4.13
ADHD+ASD	26.45	5.79	1.38
LD only	1.93		
ID only	1.38		
Unknown	1.93		

N = 363

### Development of Parenting Resilience Elements Scale (PREQ)

A pool of 44 candidate items was developed according to the qualitative analysis of parenting resilience [17]. This pool was reviewed by co-authors and clinicians and reduced to 34 items. The revised pool was used with 40 mothers of children with DD. We excluded and expanded the items based on analysis of this data, resulting in a final pool of 29 items. Each item was scored on a seven-point Likert scale from 1 (strongly disagree) to 7 (strongly agree).

### Measures

We used 12 items from a Japanese version of a general health questionnaire (GHQ) to evaluate mental health [23, 24]. Items were rated on four-point scales with labels varying across items. The sum of scores (range = 0–36) was used for the analysis. Higher scores indicated greater psychological distress. Cronbach’s alpha for GHQ showed good internal consistency in the current sample (alpha = .83, n = 313).

A Japanese version of the Center for Epidemiological Studies Depression Scale (CES-D) was used to assess depressive symptoms [25, 26]. The CES-D comprises 20 items that rate the frequency of depressive symptoms from 0 (never or one day a week) to 3 (more than five days a week). We used the sum score of 20 items (range = 0–60) for the analysis. In the current sample, this CES-D showed good internal consistency (alpha = .85).

The Parenting Scale (PS) comprised 30 items measuring ineffective discipline style [27]. The Japanese version of the PS has two factors: overreactivity (10 items) and laxness (eight items) [22]. In this study, we examined parenting behavior using items of overreactivity in the Japanese version of the PS. Participants rated each item using a seven-point Likert scale. The sum score was used for analysis. Overreactivity had strong internal consistency in the current sample (alpha = .90).

The child’s behavior was assessed using a Japanese version of the strength and difficulties questionnaire (SDQ) [28, 29]. The SDQ contains 25 items divided into five subscales: hyperactivity-inattention, emotional symptoms, peer problems, conduct problems, and prosocial behavior. The first four subscales represent negative aspects of child’s behavior and are summed to obtain a total difficulties score. The total difficulties score was used for the analysis. Participants rated their child’s behavior using a three-point Likert scale from 0 (not true) to 2 (certainly true). The total difficulties score had good internal consistency in the current sample (alpha = .83, n = 313).

### Statistics

Statistics analysis was conducted using M-plus version 7.11 for confirmatory factor analysis [30] and R version 3.01 [31] for other analyses.

## Results

### Preliminary analysis

Three items were removed because more than 40% of participants selected the highest or lowest response, which showed the ceiling/floor effect. The remaining 26 items ranged in skewness from −1.29 to .72 and in kurtosis from −.84 to 3.01, indicating proximity to a normal distribution.

### Exploratory factor analysis

Exploratory factor analysis was conducted on the 26 items using the maximum likelihood estimation method with oblique promax rotation. The number of factors was determined by parallel analysis. We then extracted three factors, as the eigenvalues from sample data were larger than the average of 1000 sets of eigenvalues from random matrices (eigenvalues of the first to the fourth factor: 7.95, 2.77, 1.90 and 1.25 from the sample data; 1.57, 1.48, 1.41, and 1.36 from the random data). In addition, the four factor solution was examined, in which the fourth factor included only two items with factor loading greater than ±.45 and which was not interpretable. Thus, we considered that the three factor solution was appropriate.

We excluded 10 items with loading of less than ±.45 in the result of the three factor solution. Exploratory factor analysis was performed again on the remaining 16 items (Table 3). The three factors accounted for 18%, 16%, and 14% of the variance, respectively. We labeled the three factors as “knowledge of the child’s characteristics,” “perceived social supports,” and “positive perceptions of parenting.”

**Table 3:** Results of exploratory and confirmatory factor analysis.

	Factor lording			
	1	2	3	CFA
**Factor 1: knowledge of the child’s characteristics**				
I know what my child is not good at.	.84	.00	-.10	.75
I know what my child will do in the future.	.77	-.06	-.11	.68
I can figure out the reason behind my child’s trouble.	.63	-.02	.02	.65
I’m aware of my child’s traits.	.63	.04	.12	.73
I have better knowledge of children’s behavior and traits than others.	.50	.16	-.04	.53
I know what my child is best suited for.(e.g., school subjects, play, and jobs).	.46	-.06	.27	.65
**Factor 2: perceived social supports**				
I have someone who I can talk to about child-raising.	-.05	.83	-.03	.79
I have someone who I can trust my child with.	.04	.80	.02	.83
I’m worried about raising my child without anyone’s opinion.	-.07	-.68	.04	-.66
There is someone who helps my child when he/she is in trouble.	-.02	.64	.05	.67
I have no choice but to raise my child all alone.	.05	-.60	.00	-.56
There are people who would help my child in the future.	-.02	.57	.01	.55
**Factor 3: positive perception of parenting**				
I value interactions with my child.	-.04	-.07	.85	.80
My child makes me feel energized.	-.08	.11	.68	.71
I enjoy talking to and playing with my child.	-.03	.05	.78	.81
I can do anything for my child that he needs.	.04	-.07	.63	.61
Inter factor correlation	1	2	3	
1	-	.30	.50	
2		-	.38	
3			-	

### Confirmatory factor analysis

Based on exploratory factor analysis, we defined a model where each item only loaded onto the appropriate factor, and three factors were allowed to correlate. Confirmatory factor analysis was performed on the model. Fit indices showed an acceptable model fit (CFI = .917, TLI = .902, RMSEA = .070, SRMR = .055) [32]. However, the chi-squared statistic suggested a poor model fit (*χ* ^*2*^(101) = 255.084, *p* < .001), as the statistic is sensitive to sample size and tends to reject the model in a large sample.

### Reliability

Cronbach’s alpha was computed for each factor, in which items with negative factor loading were reverse-scored. Cronbach’s alphas were .81 for “knowledge of the child’s characteristics,” .84 for “perceived social supports,” and .81 for “positive perceptions of parenting.” These findings showed good internal consistency of PREQ subscales.

### Validity

We used sum scores of subscales and the total score of PREQ for further analysis. Table 4 shows the correlation of PREQ scores with PS overreactivity, CES-D, GHQ, and SDQ total difficulties score. Consistent with a definition in which parenting resilience reduces overreactive parenting and psychological distress, PREQ scores were negatively correlated with PS overreactivity, CES-D, and GHQ.

**Table 4:** The relationship of subscales and total score of PREQ with parenting style, psychological distress and the child’s behavior.

	PS	CES-D	GHQ	SDQ
knowledge of the child’s characteristics	-.27***	-.22***	-.18***	-.07***
perceived social supports	-.19***	-.44***	-.39***	-.18***
positive perception of parenting	-.37***	-.31***	-.21***	-.13***
Total score	-.35***	-.47***	-.39***	-.18***

* < .05,

** < .01,

*** < .001

PS: parenting scale (overreactivity), CES-D: center for epidemiologic studies depression scale, GHQ: general health questionnaire-12, SDQ: strength and difficulties questionnaire (total difficulties score)

To clarify the association of PREQ subscales with parenting behavior and depressive symptoms, multiple regression analysis was conducted with PS overreactivity and CES-D as dependent variables (Table 5). PS overreactivity and CES-D were significantly predicted by the model including PREQ subscales, GHQ, and SDQ total difficulties score (*F* (5,307) = 14.88, *p* < .001; *F* (5,307) = 124.90, *p* < .001). In the model, “knowledge of the child’s characteristics” and “positive perceptions of parenting” were significantly associated with PS overreactivity, whereas “perceived social supports” and “positive perceptions of parenting” significantly predicted CES-D. SDQ total difficulties score and GHQ were significantly associated with both PS overreactivity and CES-D.

**Table 5:** Results of multiple regression analysis of PS and CES-D

	PS		CES-D	
	β	*t*	β	*t*
GHQ	.16	2.87***	.70	19.30***
SDQ total difficulties score	.11	2.04***	.09	2.70***
knowledge of the child’s characteristics	-.13	-2.29***	-.02	-.54***
perceived social supports	.00	.09***	-.11	-2.96***
positive perception of parenting	-.27	-4.68***	-.11	-3.08***
R^2^	.20***		.67***	
Adjusted R^2^	.18***		.67***	

* < .05,

** < .01,

*** < .001

PS: parenting scale (overreactivity), CES-D: center for epidemiologic studies depression scale, GHQ: general health questionnaire, SDQ: strength and difficulties questionnaire

### The relationship between PREQ factor and the characteristics

To examine the difference among diagnostic groups, we performed a univariate analyses of variance (ANOVAs) with groups (ADHD, ASD, ADHD+ASD, other) on each sum score of PREQ subscale (Table 6). We applied type III sum of squares because of the unequaled group size. There were no significant main effects of groups in subscales (knowledge of the child’s characteristics: *F*(3,359) = 0.41, *p* = .74; perceived social supports: *F*(3,359) = 0.44, *p* = .73; positive perception of parenting: *F*(3,359) = 1.27, *p* = .28). In addition, correlational analyses were performed between child’s age and sum score of each subscale to investigate the effect of development. Child’s age was significantly correlated with sum scores of perceived social supports (*r* = -.13, *p* < .05) and positive perception of parenting (*r* = -.11, *p* < .05). The correlation between child’s age and the sum score of knowledge of the child’s characteristics was not significant (*r* = -.04, *p* = .49). Furthermore, we examined the relation between age of child’s diagnosis and sum score of each subscales. The age of child’s diagnosis significantly correlated with sum scores of perceived social support (*r* = -.17, *p* < .01) and positive perception of parenting (*r* = -.15, *p* < .01). No significant correlation was found between the age of child’s diagnosis and the sum score of knowledge of the child’s characteristics (*r* = -.02, *p* = .69).

**Table 6:** Means (standard deviations) of sum scores of each subscale in diagnostic groups (N = 363).

	ADHD	ASD	ADHD+ASD	other
knowledge of the child’s characteristics	31.11 (4.27)	30.51 (4.55)	30.67 (4.24)	31.05 (2.84)
perceived social supports	29.69 (7.89)	30.56 (6.10)	29.80 (6.50)	30.50 (6.27)
positive perception of parenting	20.69 (4.05)	21.55 (3.62)	21.13 (3.34)	21.80 (3.24)

## Discussion

We developed the parenting resilience questionnaire (PREQ) to measure resiliency for rearing children with DD in primary caregivers and further evaluated its psychometric properties. Exploratory factor analysis showed a three-factor structure for parenting resilience, i.e., “knowledge of the child’s characteristics,” “perceived social supports,” and “positive perceptions of parenting.” This structure was supported by confirmatory factor analysis, and internal consistency of each factor was good. Corresponding to the definition of parenting resilience, the scores of the three factors were negatively correlated with psychological distress and overreactive parenting behavior.

The first factor of the PREQ was “knowledge of the child’s characteristics.” This factor includes items reflecting the perception of having the requisite knowledge and skills for rearing children with DD. Parental training intervention, designed to teach knowledge and skills in rearing children, was reported to reduce parental stress [33]. In the previous study, mothers of children with ADHD who perceived that they had control over child behavior tended to have lower psychological distress levels [12]. In line with these studies, “knowledge of the child’s characteristics” was negatively correlated with psychological distress. Importantly, multiple regression analysis revealed that higher scores of this factor were associated with lower levels of overreactive parenting behavior. We suggested that caregiver’s appropriate parenting behavior derives from prediction according to knowledge about their children.

The second factor of the PREQ was “perceived social supports.” Previous studies showed that a lack of perceived social support caused psychological distress in mothers of children with ASD [9]. Consistent with previous studies, “perceived social supports” predicted depressive symptoms. The quality of social supports can be assessed in various ways, e.g., network size [10], valence [10], and kinds of source [34]. These measures have different effects on the psychological distress of caregivers of children with DD. The factor “perceived social supports” is considered to reflect the total effect of social supports.

The third factor of the PREQ was “positive perceptions of parenting.” This factor comprised items related to pleasure and happiness in rearing the child and the acceptance of the parental role. Higher scores of this factor were associated with lower levels of both depressive symptoms and overreactive parenting behavior. Previous studies showed that positive perceptions allow caregivers of children with ID to positively reframe difficulties and problems relative to their child [35]. The coping strategy of positive reframing reportedly decreased depressive symptoms in parents of children with ASD [11]. We suggested that “positive perceptions of parenting” of PREQ is close in concept to the idea of “positive perceptions” reported by Hastings and Taunt [8]. Therefore, “positive perceptions of parenting” is considered to play an important role for caregivers in coping with child problems and adapting to challenges and difficulties in rearing children with DD.

The correlational coefficients between the SDQ total difficulties score and PREQ scores were relatively low, i.e., “knowledge of the child’s characteristics” was not significantly correlated with the SDQ total difficulties score. These results suggested that PREQ was able to measure caregivers’ resiliency independent of child behavior problems. On the other hand, multiple regression analysis showed that sum scores of PREQ were associated with decreases in the depressive symptoms and the overreactive parenting, which suggested that caregivers with high PREQ scores behave appropriately with respect to their children. Thus, it is possible that symptoms of their children are improved over time by treatment of caregivers with high PREQ scores. Longitudinal studies such as those concerning effectiveness of parental training will facilitate exploration of the relationship between PREQ and improvement of child behavior in the future.

In a previous study, we proposed a model of parenting resilience comprising five categories [17] using M-GTA [21]. PREQ was developed according to the model used in the previous study [17], but it comprised only three factors. This structural difference stems from a methodological difference; M-GTA revealed dynamic process, whereas the methodology in this study could not explicitly distinguish temporal characteristics. Thus, items of “foresight” [17] belonged to “knowledge of the child’s characteristics” and “perceived social supports” in the PREQ factor structure. We excluded the items that had been predicted to be included in “self-efficacy,” e.g., “My efforts have contributed partly to children’s growth,” “My child-raising practices have influenced my child positively,” and “There is nothing I can do to help my child when he/she is in trouble (reverse)”, because they did not form another factors and were low factor loadings in the three factor solution. The “self-efficacy” is considered to be shaped as the results of past parenting. Hence, we suggest that some items in “knowledge of characteristics” are associated with “self-efficacy.” In addition, in the cross-cultural study, the Japanese participants tended to have self-criticism and were careful in not expressing their superior skill [36], which might distort to self-rate the items relevant to “self-efficacy.” Therefore, we expected that extra items measuring “self-efficacy” were added to PREQ when applying to other culture.

It is possible that a parent’s personality and traits interact with parenting resilience. Ekas et al. [34] reported that parental optimism was a mediator of the effect of social supports on well-being and psychological distress. In a previous study, a higher level of the broader autistic phenotype (BAP) was related to lower social supports and inappropriate coping [37]. Thus, we should interpret PREQ scores from several aspects based on clinical observations.

Several frameworks of resilience proposed that resilience elements were acquired over time [14]. Thus, we also assumed that caregivers acquired elements of parenting resilience by experience of rearing children and clinical intervention [17,20]. Contrary to our prediction, positive correlations between child’s age and the sum scores of PREQ subscales were not found in this study. Moreover, child’s age was negatively correlated with sum scores of perceived social supports and positive perception of parenting. On the other hand, significant correlations was found between the age of child’s diagnosis and sum scores of perceived social supports as well as positive perception of parenting, which suggested that delay in diagnosis and treatments might cause social isolation and disinclination for parenting. Thus, the effect of child’s age was confounded by the influence of age of child’s diagnosis since child’s age was correlated with the age of child’s diagnosis in our sample (*r* = .60, *p* = .00). In addition, there was a limit for clarifying the developmental changes in a cross-sectional study. It is important to examine the process of acquiring the elements of parenting resilience. Hence, we expected that acquisition of elements of parenting resilience will be examined by a long-term cohort study.

Certain issues relating to our sample should be considered when applying PREQ to clinical practice or research. First, the PREQ was developed and evaluated only for a Japanese population. As mentioned above, the construct of PREQ might be influenced by Japanese culture. Besides the self-rating of Japanese, for example, the religious factor was considered to be a source of resilience in Europe/North America [11, 18], whereas most Japanese are known as non-religious, and this factor was not reported in the previous study [17]. Therefore, further studies in a cross-cultural population will be required to evaluate PREQ items. Next, participants may have been biased, as they were recruited directly from medical institutes. Most participants had positive relationships with their medical institutes. The difficulties of their children were considered to be so severe that the children needed medication and medical intervention. These characteristics might affect the results of this study. Furthermore, we merged diagnostic groups into one group. Although the different sum scores of PREQ subscales among diagnostic groups (i.e., ADHD, ASD, ADHD+ASD, and other) were not found in this study, it was possible that the constructs of PREQ were inconsistent among diagnostic groups. When considering elements of parenting resilience for one of the diagnostic group, elements specific to a diagnostic group (e.g., skills of parenting) may be postulated. Finally, we only analyzed data from mothers of children with DD. The construct of parenting resilience can be influenced by sex and parental roles. Therefore, the PREQ difference from mothers to fathers (and other caregivers) should be considered when applying them. Further studies are required to examine the construct of parenting resilience in fathers and other caregivers.

In summary, a newly developed PREQ is interpretable as a measure of the degree to which mothers possess elements of resilience in rearing children with DD. PREQ allows assessment of resiliency of mothers of children with DD, which may lead to early intervention for primary caregivers before they are deeply depressed by child problems. Moreover, using the PREQ, we hope that future studies investigate the relationship between children and caregivers and evaluate the effects of interventions.

## Supporting Information

S1 File Dataset for factor analyses. (CSV)

S2 File Dataset for correlational analyses. (CSV)
