# Correction: Development and Evaluation of a Parenting Resilience Elements Questionnaire (PREQ) Measuring Resiliency in Rearing Children with Developmental Disorders

**DOI:** 10.1371/journal.pone.0146090

**Published:** 2015-12-29

**Authors:** The PLOS ONE Staff

Some of the indicated p-values in Tables 4 and 5 are incorrect. The publisher apologizes for the errors. Please see the corrected Tables 4 and 5 here.

**Table 4:** The relationship of subscales and total score of PREQ with parenting style, psychological distress and the child’s behavior.

	PS	CES-D	GHQ	SDQ
knowledge of the child’s characteristics	-.27***	-.22***	-.18**	-.07
perceived social supports	-.19***	-.44***	-.39***	-.18***
positive perception of parenting	-.37***	-.31***	-.21***	-.13*
Total score	-.35***	-.47***	-.39***	-.18***

* < .05

** < .01

*** < .001

PS: parenting scale (overreactivity), CES-D: center for epidemiologic studies depression scale, GHQ: general health questionnaire-12, SDQ: strength and difficulties questionnaire (total difficulties score)

**Table 5:** Results of multiple regression analysis of PS and CES-D

	PS		CES-D	
	β	*t*	β	*t*
GHQ	.16	2.87***	.70	19.30***
SDQ total difficulties score	.11	2.04*	.09	2.70**
knowledge of the child’s characteristics	-.13	-2.29*	-.02	-.54
perceived social supports	.00	.09	-.11	-2.96**
positive perception of parenting	-.27	-4.68***	-.11	-3.08**
R^2^	.20***		.67***	
Adjusted R^2^	.18		.67	

* < .05

** < .01

*** < .001

PS: parenting scale (overreactivity), CES-D: center for epidemiologic studies depression scale, GHQ: general health questionnaire, SDQ: strength and difficulties questionnaire
